# Increasing burden of community-acquired pneumonia leading to hospitalisation, 1998–2014

**DOI:** 10.1136/thoraxjnl-2015-207688

**Published:** 2016-02-17

**Authors:** T Phuong Quan, Nicola J Fawcett, John M Wrightson, John Finney, David Wyllie, Katie Jeffery, Nicola Jones, Brian Shine, Lorraine Clarke, Derrick Crook, A Sarah Walker, Timothy E A Peto

**Affiliations:** 1 NIHR Oxford Biomedical Research Centre, Oxford, UK; 2 Nuffield Department of Medicine, University of Oxford, Oxford, UK; 3 Oxford University Hospitals NHS Foundation Trust, Oxford, UK

**Keywords:** Pneumonia

## Abstract

**Background:**

Community-acquired pneumonia (CAP) is a major cause of mortality and morbidity in many countries but few recent large-scale studies have examined trends in its incidence.

**Methods:**

Incidence of CAP leading to hospitalisation in one UK region (Oxfordshire) was calculated over calendar time using routinely collected diagnostic codes, and modelled using piecewise-linear Poisson regression. Further models considered other related diagnoses, typical administrative outcomes, and blood and microbiology test results at admission to determine whether CAP trends could be explained by changes in case-mix, coding practices or admission procedures.

**Results:**

CAP increased by 4.2%/year (95% CI 3.6 to 4.8) from 1998 to 2008, and subsequently much faster at 8.8%/year (95% CI 7.8 to 9.7) from 2009 to 2014. Pneumonia-related conditions also increased significantly over this period. Length of stay and 30-day mortality decreased slightly in later years, but the proportions with abnormal neutrophils, urea and C reactive protein (CRP) did not change (p>0.2). The proportion with severely abnormal CRP (>100 mg/L) decreased slightly in later years. Trends were similar in all age groups. *Streptococcus pneumoniae* was the most common causative organism found; however other organisms, particularly *Enterobacteriaceae*, increased in incidence over the study period (p<0.001).

**Conclusions:**

Hospitalisations for CAP have been increasing rapidly in Oxfordshire, particularly since 2008. There is little evidence that this is due only to changes in pneumonia coding, an ageing population or patients with substantially less severe disease being admitted more frequently. Healthcare planning to address potential further increases in admissions and consequent antibiotic prescribing should be a priority.

**Key messages**

**What is the key question?**

- UK hospitalisations for pneumonia were found to be increasing more than expected between 1997 and 2009, but what is happening now, and is there anything we should be worrying about?

**What is the bottom line?**

- There is an alarming ongoing increase in pneumonia hospitalisations (at least in Oxfordshire), as well as changes in bacterial aetiology which have implications for antibiotic prescribing.

**Why read on?**

- We show that the increase is not just due to changes in population, documentation or admission practices by triangulating frequently used but arguably subjective data such as diagnostic codes against more reliably objective data such as laboratory tests.

Community-acquired pneumonia (CAP) is a major cause of mortality and morbidity worldwide, particularly among people over 65 years of age,^1^ ^2^ and is a key driver for antibiotic prescribing. Given the ageing population in high-income countries and concerns over antibiotic resistance, it is surprising how little research has investigated the burden CAP places on our hospitals. For example, the two large-scale studies investigating pneumonia incidence in English hospitals^3^ ^4^ showed a marked increase in admissions from 1997 to 2005 and 2002 to 2009 respectively, but no studies have reported more recent trends.

Beyond an actual increase in disease, the increases reported have many potential explanations, including changes in case-mix, improvements in diagnostic coding, changes in preferred codes (eg, ‘sepsis’ rather than ‘pneumonia’^5^) or changing admission practices, for example, admitting lower-severity cases. However, most research to date has used only administrative coding data sets, such as the UK Hospital Episode Statistics or the US National (Nationwide) Inpatient Sample, which alone are unable to assess disease severity at presentation to hospital, and hence whether or not this affects admission practices.

We therefore used the Infections in Oxfordshire Research Database (IORD)^6^ to investigate the hypothesis that changes in incidence of hospitalisations for CAP in Oxfordshire adults over the past 16 years could be explained by changes in case-mix, coding practices, disease severity at admission or bacterial aetiology.

## Methods

IORD contains all admissions to the Oxford University Hospitals NHS Foundation Trust from April 1997 to date, linked with microbiology and biochemistry/haematology laboratory results. The four Trust hospitals provide all acute care and all microbiology and pathology services in the region (∼600 000 individuals). Out-of-hospital mortality is determined from a national information system into which UK-wide deaths are recorded.

This analysis considered UK financial years (April–March) ending 1998–2014 inclusive (avoiding splitting the winter peak across 2 years) and included only patients ≥18 years at admission. To restrict to community-acquired cases, we only included patients with a primary diagnostic code of pneumonia admitted to specialties where CAP would normally present, that is, acute general medicine (300), infectious diseases (350), emergency department (180), respiratory medicine (340) and geriatric medicine (430). If an admission contained multiple consultant episodes, only the first episode's codes were used. Over the study period, these specialties accounted for 17 489/18 785 (93%) adult pneumonia admissions. All primary diagnostic codes in these specialties were grouped into diagnostically distinct categories by an acute medicine physician (referring to the Clinical Classifications software^7^), and reviewed by an infectious diseases specialist (see online supplementary material).

We also excluded all day cases (where a patient temporarily requires a bed but is not expected to stay overnight (173/17 489 (1%) adult pneumonia admissions)) as day cases had been recorded in different ways in different years. Trends in annual incidence were modelled using Poisson regression, with financial year as the only explanatory variable, and allowing a change in trend where this significantly improved model fit (see online supplementary methods; in brief, the deviance goodness-of-fit statistic from models allowing a change in trend at each calendar year between 2000 and 2012 inclusive were compared,^8^ and the lowest deviance two-trend model compared with the model with a single trend using a χ^2^ test). To assess whether any changes in pneumonia incidence merely reflected changes in admissions in general, we separately modelled adult admissions (to the same specialties) for other common infections, all non-pneumonic infections and all non-infectious causes, comparing rates across different diagnosis groups using stacked regression.

In sensitivity analyses, we modelled only the first pneumonia episode per person per year, as well as only the first pneumonia episode per person recorded since April 1997 (to determine if changes were driven by increases/decreases in readmissions rather than actual disease). We also separately considered cases of pneumonia where the patient had not been in hospital in the preceding 14 days, to exclude the possibility of them having acquired their pneumonia during a previous hospital admission.

### Case-mix and population growth

Pneumonia disproportionately affects older people so to control for this we standardised admissions for age (and sex) for each year against the 1998 Oxfordshire population, and also adjusted for overall population size. Population estimates were obtained from the UK Office for National Statistics.^9^

Comorbidities also increase with age, and are often controlled for by calculating the Charlson comorbidity score^10^ for each episode. However, the completeness of secondary diagnostic codes has substantially improved over time^11^ (see online supplementary figure S1), and so the Charlson comorbidity score cannot be relied upon to measure comorbidities consistently over long time periods (see online supplementary figure S2).

### Diagnostic coding

Under clinical uncertainty or multiple diagnoses, the choice of primary diagnostic code depends on many factors, including documentation quality, the clinical coder's experience,^12^ the higher monetary tariff paid for certain codes, and quality improvement projects that may encourage the use of certain codes. Therefore, pneumonia cases may potentially be mislabelled as another lower respiratory disorder or vice versa, so we also modelled the incidence over time of likely alternative diagnoses, namely COPD, other lower respiratory tract infections, sepsis and non-specific viral infection (see online supplementary material). ‘Diagnostic-code- switching’ would be suggested if these incidence trends were opposite to that of pneumonia.

### Disease severity and aetiology

To assess whether changes in disease severity of those being admitted to hospital could be contributing to changes in pneumonia incidence, we first considered those measures available in other administrative data sets, namely the outcomes length of stay, 30-day all-cause mortality and 30-day readmission. The major limitation of using such outcomes to assess CAP severity at presentation is that they are influenced by numerous factors during admission, for example, delayed discharge for social reasons or administrative changes in bed policy. As more robust measures of severity we therefore also modelled the median C reactive protein (CRP), urea and absolute neutrophil count (closest value within ±2 days of admission), as well as the proportion of these test results falling outside prescribed thresholds (CRP: >20 and >100 mg/L,^13^ urea: >7 mmol/L,^14^ neutrophils: <2 or >7 ×10^9^/L (hospital laboratory reference range)). Results from the Horton laboratory (which closed in October 2003 when all testing was transferred to a single site) were not available, and so only test results available for complete financial years were included, and a step-change added to the models at this time point to allow for case-mix differences between the hospitals. Test results were therefore available in 67–70% CAP admissions annually before 2004 and 90–97% thereafter.

Finally, we modelled the incidence and proportion of CAP admissions where microbiology cultures of blood or high-quality respiratory samples (ie, pleural fluid or bronchoalveolar lavage) were taken (within ±2 days of admission), and any likely causative agents found (see online supplementary material). Where there were multiple microbiological tests within the window, the earliest positive pleural fluid sample was taken where available, otherwise the earliest positive blood sample, otherwise the earliest positive bronchoalveolar lavage sample. CAP sputum samples are not routinely analysed at the hospital laboratory owing to poor reliability and so were not considered. Similarly to the haematology/biochemistry tests, microbiology results from the Horton laboratory were not available so only results available for complete financial years were included.

Trends in length of stay and median biomarkers were modelled using median (quantile) regression, and in other outcomes using Poisson regression. All analyses were conducted using Stata V.13.1.

## Results

Between April 1997 and March 2014 there were 407 774 adult admissions into the relevant specialties, comprising 195 489 individuals. Of these admissions, 17 316 were for CAP. Just over half of CAP admissions (52%) were men and this proportion did not change significantly over time (p=0.53). Median age increased over time (p<0.001) from 74 years in 1997/1998 to 80 years in 2013/2014.

**Figure 1:**
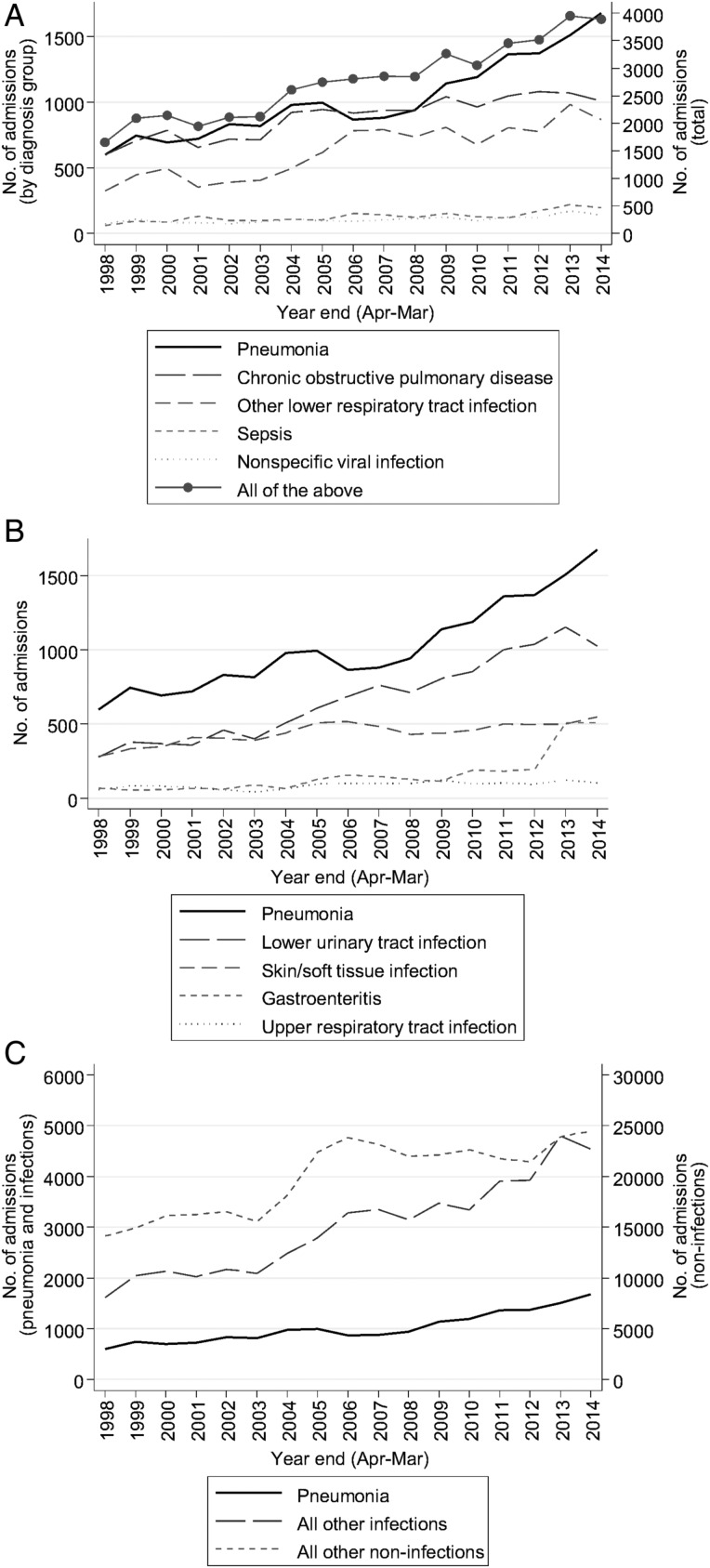
Annual admissions for specific diagnostic groups. (A) Pneumonia versus similar diagnostic conditions (B) Pneumonia versus other common infections (C) Pneumonia versus all other codes.

Raw CAP admissions rose from 598 in 1997/1998 to 1677 in 2013/2014, an overall rise of 180% (figure 1A). However, there was strong evidence (p<0.001) this had not occurred uniformly, with admissions increasing by 4.2%/year (95% CI 3.6 to 4.8) through 2007/2008, but by 8.8%/year (95% CI 7.8 to 9.7) afterwards (table 1). Faster annual incidence increases post 2008 persisted after controlling for population changes (p<0.001 vs model with no trend-change), although the absolute increases were smaller. Annual incidence increases were also significantly faster post 2008 when including only the first pneumonia episode per person per year (p<0.001), and faster post 2007 when including only the first pneumonia episode per person ever recorded (p<0.001), eliminating the possibility that readmissions were driving overall increases. Patterns remained similar when excluding patients who had been in hospital in the preceding 14 days. Very few admissions (<30/year) were recorded as coming from nursing homes.

**Table 1:** Annual trends in incidence of admissions for pneumonia and other related diagnoses (to Acute General Medicine and related specialties)

	Initial % change per year (95% CI)	Year of rate-change*	Subsequent % change per year (95% CI)	Direction of change†	Estimate in 2014‡
Pneumonia admissions					
Unadjusted	4.2 (3.6 to 4.8)	2008	8.8 (7.8 to 9.7)	↑ ↑	1668
Standardised for age, sex and population size	2.0 (1.4 to 2.6)	2008	5.1 (4.2 to 6.1)	↑ ↑	n/a
Excluding readmissions in same (financial) year	4.1 (3.5 to 4.7)	2008	8.2 (7.2 to 9.1)	↑ ↑	1528
Excluding readmissions at any later date	3.0 (2.3 to 3.7)	2007	6.9 (6.1 to 7.8)	↑ ↑	1318
Excluding those discharged from our hospitals in the previous 14 days	3.6 (3.0 to 4.3)	2008	8.4 (7.5 to 9.4)	↑ ↑	1425
Possible alternative diagnoses					
COPD	5.5 (4.6 to 6.5)	2005	1.9 (1.3 to 2.5)	↑ ↑	1076
Other lower respiratory tract infections	9.1 (8.2 to 10.0)	2007	2.9 (2.0 to 3.9)	↑ ↑	885
Sepsis	4.4 (3.1 to 5.6)	2011	13.5 (8.0 to 19.3)	↑ ↑	217
Non-specific viral infection	2.8 (1.3 to 4.2)	2010	8.7 (4.2 to 13.3)	↑ ↑	156
All pneumonia-like diagnoses	5.7 (5.1 to 6.2)	2005	4.6 (4.3 to 5.0)	↑ ↑	3926
Other common infections					
Lower urinary tract infections	9.5 (9.0 to 10.0)	2012	−0.6 (−3.9 to 2.7)	↑ ↔	1067
Skin and soft tissue infections	6.5 (5.1 to 7.9)	2005	0.7 (−0.2 to 1.6)	↑ ↔	502
Upper respiratory tract infections	3.9 (2.8 to 5.0)	–		↑	115
Gastroenteritis	10.1 (8.7 to 11.4)	2011	45.4 (40.0 to 51.0)	↑ ↑	564
Overall admissions					
All admissions	6.2 (6.0 to 6.4)	2006	1.6 (1.5 to 1.8)	↑ ↑	29 438
All infections (excluding pneumonia)	7.1 (6.6 to 7.6)	2006	5.7 (5.3 to 6.1)	↑ ↑	4601
All non-infections	6.2 (6.1 to 6.4)	2006	0.7 (0.5 to 0.8)	↑ ↑	23 274

All trends modelled using piecewise linear Poisson regression. See Methods for included specialties.

*Allowing change in trend over time if significant improvement over linear trend alone (p<0.05), with year of the change point chosen by profile likelihood^8^ (see Methods).

†Single arrows represent direction of change over the whole time period; paired arrows represent direction and relative size of change in the periods before and after the year when data suggested trend in rates changed (as in *).

‡Number of admissions, as estimated by the best-fitting model as presented in the table.

Admissions for other conditions which might be alternatively diagnosed as pneumonia also increased significantly during this time (figure 1A and table 1). However, in contrast to pneumonia, increases in COPD and in other lower respiratory infections were faster before 2005 and 2007, respectively, than subsequently, suggesting some diagnosis-code switching could be occurring. Comparing current incidence rates, CAP incidence rose significantly faster than COPD and other lower respiratory infections (p<0.001 and p=0.001, respectively), but there was no evidence of difference in trends compared with sepsis and non-specific viral infections (p=0.34 and p=0.98, respectively). CAP incidence also rose significantly faster compared with all the above conditions grouped together (p<0.001).

Admissions for other common infections also increased during the period (figure 1B), with lower urinary tract infections, skin and soft tissue infections, and upper respiratory infections all currently rising at significantly lower rates (p<0.001) than pneumonia, while gastroenteritis is currently rising significantly faster (p<0.001). However, lower urinary tract infections and gastroenteritis had very late trend-change points (2012 and 2011, respectively), and prior to this there was no evidence that either diagnosis was rising at a different rate to post-2008 pneumonia (p=0.45 and p=0.40, respectively).

Overall admissions (into the relevant specialties) increased, though with a current trend significantly slower than CAP (p<0.001). Similarly, admissions for all non-pneumonic infections and for all non-infections also increased over the study period (figure 1C), but with current trends significantly slower than CAP (p=0.02 and p<0.001, respectively).

**Figure 2:**
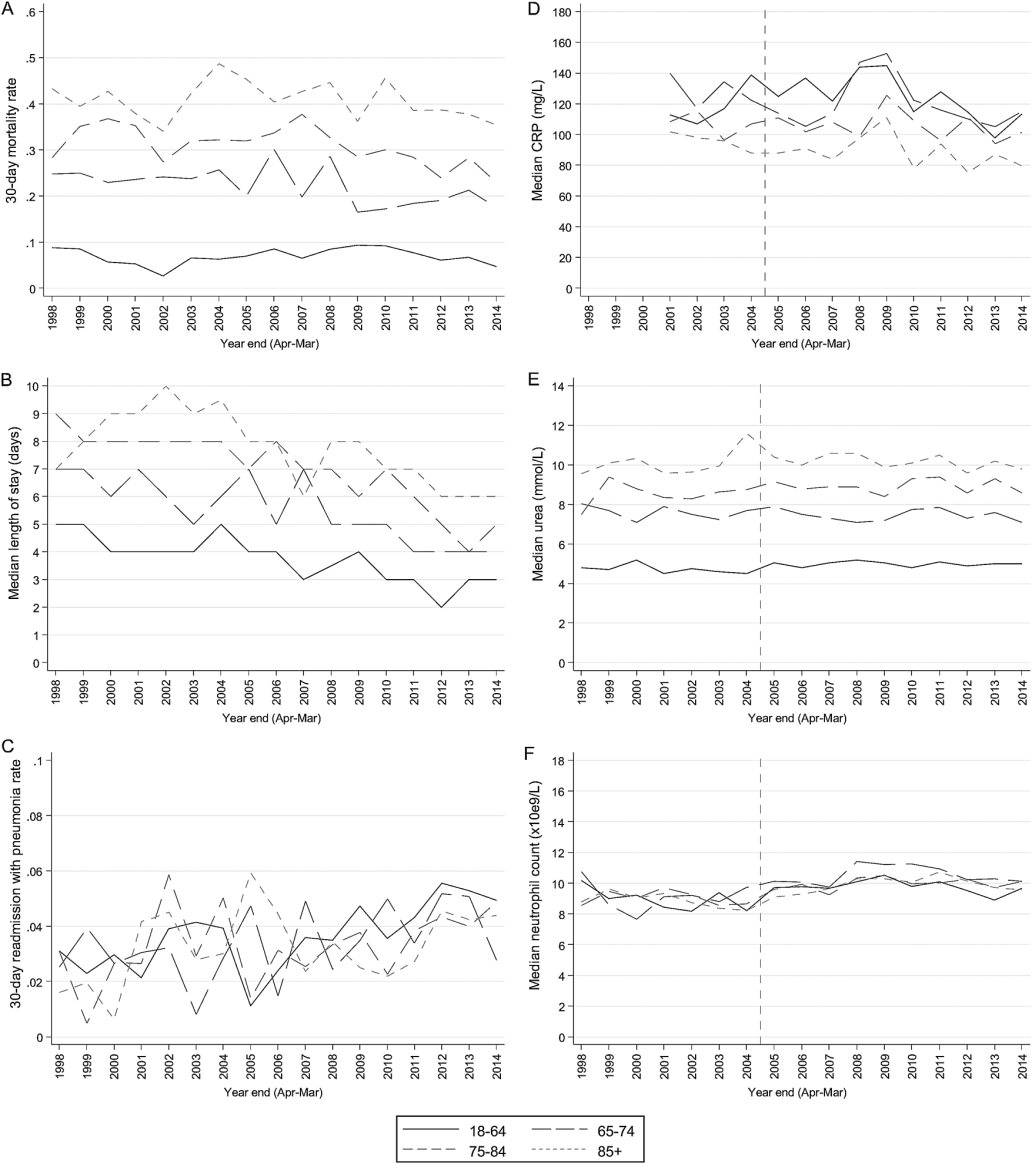
Administrative outcomes and admission biomarkers for all community-acquired pneumonia admissions. (A) 30-day all-cause mortality rate (B) median length of stay (C) 30-day readmission rate (with pneumonia) post discharge (D) median C reactive protein (CRP) at admission (E) median urea at admission (F) median neutrophil count at admission. Vertical line represents step-change in availability of laboratory test results (see Methods).

Considering administrative outcomes of adult CAP admissions, 30-day all-cause mortality ranged from 22% to 29% over the study, increasing by 1.8%/year from 1998 to 2007 then decreasing by 3.1%/year (table 2). Median length of stay stayed constant at ∼6 days from 1998 to 2005, then decreased by 0.25 days/year. Trends were similar in all age groups (figure 2). This suggests the threshold for admitting patients with CAP could have lowered in recent years, and/or care had improved. However, while 2–5% of patients were readmitted with pneumonia (as primary diagnosis) within 30 days of discharge, this significantly increased over the study, and similarly, all-cause readmissions increased from 2002 onwards. Competing-risks regression^15^ also confirmed that readmissions were increasing significantly faster than deaths post discharge (sub-HR=1.07/year (1.05 to 1.09)).

**Table 2:** Annual trends in administrative data outcomes (in those admitted for pneumonia)

	Initial change* per year (95% CI)	Year of rate-change†	Subsequent change* per year (95% CI)	Direction of change‡	Estimate in 2014§
30-day mortality	1.8 (0.4 to 3.1)	2007	−3.1 (−4.5 to −1.7)	↑ ↓	0.22
30-day readmission					
All-cause	10.0 (4.2 to 16.1)	2002	1.5 (0.4 to 2.6)	↑ ↑	0.16
Pneumonia	3.4 (1.7 to 5.2)	−		↑	0.04
Median length of stay (days)	0.00 (−0.06 to 0.06)	2006	−0.25 (−0.30 to −0.20)	↔ ↓	4

Length of stay modelled using piecewise median regression, all other trends using piecewise linear Poisson regression.

*Percentage change in rate trend for mortality, readmission; absolute change in median length of stay.

†Allowing change in trend over time if significant improvement over linear trend alone (p<0.05), with year of the change point chosen by profile likelihood^8^ (see Methods).

‡Single arrows represent direction of change over the whole time period; paired arrows represent direction and relative size of change in the periods before and after the year when data suggested trend in rates changed (as in †).

§Proportion of admissions/median value, as estimated by the best-fitting model as presented in the table.

Considering biomarkers that should reflect disease severity at presentation more accurately than postadmission outcomes, median CRP increased slightly up to 2009 then decreased slightly thereafter (table 3); however, levels remained significantly elevated (median 94 mg/L in 2014). The proportion of results over 100 mg/L was stable up to 2009 at ∼52% then decreased by 2.3%/year. Other biomarkers were mostly stable, with median urea increasing slightly up to 2004 then remaining at ∼7.8 mmol/L, and median neutrophil counts stable up to 2011 then decreasing slightly thereafter (median 9.7×10^9^/L in 2014). The proportion of these tests which were outside relevant thresholds did not change substantially over time (p=0.21, p=0.73, respectively). Again, trends were similar in all age groups (figure 2).

**Table 3:** Annual trends in admission biomarkers (in those admitted for pneumonia)

	Initial change* per year (95% CI)	Year of rate-change†	Subsequent change* per year (95% CI)	Direction of change‡	Estimate in 2014§
Median CRP (mg/L)	2.68 (0.18 to 5.17)	2009	−4.60 (−6.48 to −2.72)	↑ ↓	94
Median urea (mmol/L)	0.12 (0.02 to 0.21)	2004	−0.01 (−0.05 to 0.03)	↑ ↔	7.8
Median neutrophils (×10^9^/L)	0.04 (−0.02 to 0.10)	2011	−0.16 (−0.28 to −0.04)	↔ ↓	9.7
Proportion CRP >20 mg/L	−0.1 (−0.8 to 0.6)	–		↔	0.84
Proportion CRP >100 mg/L	1.0 (−1.1 to 3.1)	2009	−2.3 (−3.8 to −0.7)	↔ ↓	0.48
Proportion urea >7 mmol/L	0.5 (−0.3 to 1.3)	–		↔	0.58
Proportion neutrophils <2 or >7×10^9^/L	−0.1 (−0.8 to 0.6)	–		↔	0.75

Median values modelled using piecewise median regression, all other trends using piecewise linear Poisson regression.

*Absolute change in median values, percentage change in rate trend for proportions outside thresholds (out of those which had a test).

†Allowing change in trend over time if significant improvement over linear trend alone (p<0.05), with year of the change point chosen by profile likelihood^8^ (see Methods).

‡Single arrows represent direction of change over the whole time period; paired arrows represent direction and relative size of change in the periods before and after the year when data suggested trend in rates changed (as in †).

§Proportion of admissions/median value, as estimated by the best-fitting model as presented in the table.

CRP, C reactive protein.

**Figure 3:**
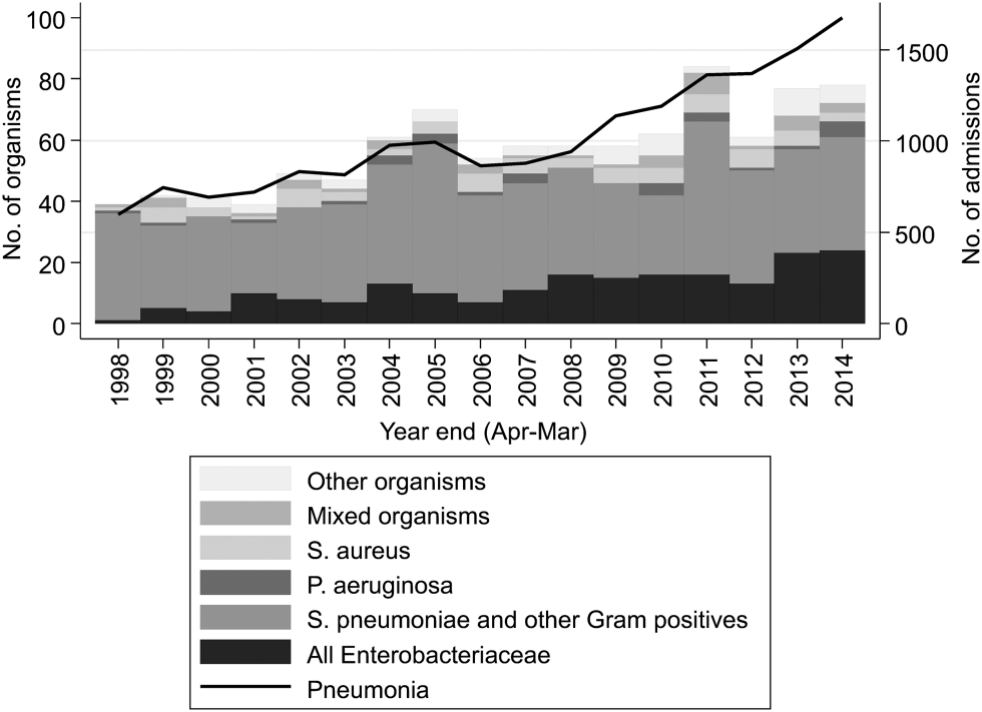
Microbiologically confirmed community-acquired pneumonia spells by causative organism (taken within 2 days of admission).

Between 53% and 69% of CAP admissions annually had blood or high-quality respiratory cultures performed, with this proportion decreasing over time (table 4), as did the proportion with a *positive* culture result. However, because of the overall increase in CAP, the absolute numbers of CAP admissions with these cultures taken, and with positive culture results, increased over time. The most common organism overall was *Streptococcus pneumoniae* (48% of cases where a causative organism was found), followed by *Escherichia coli* (11%) and *Staphylococcus aureus* (7%). Incidence of CAP primarily associated with *Enterobacteriaceae* increased significantly over the study period (*E. coli*: 9.8%/year, p<0.001; *Klebsiella* spp: 12.3%/year, p=0.001; other *Enterobacteriaceae*: 10.6%/year, p=0.001) (figure 3), as did cases associated with anaerobes/*Streptococcus ‘milleri’* group bacteria (13.6%/year, p=0.01). Incidence of other traditional CAP-related organisms did not change significantly (*S. pneumoniae*: p=0.63; *S. aureus*: p=0.84; *Haemophilus influenzae*: p=0.50; *Pseudomonas aeruginosa*: p=0.25; other Gram-positives: p=0.25; other Gram-negatives: p=0.22). The increasing incidence of *Enterobacteriaceae*­-associated CAP persisted even when excluding individuals who had been in hospital in the previous 14 days (see online supplementary figure S3); and the median (IQR) time from admission to specimen collection was −0.7 (−4.1 to 4.0) hours, suggesting this is highly unlikely to be driven by misclassified hospital-acquired pneumonia.

**Table 4:** Annual trends in microbiology results (in those admitted for pneumonia)

	Initial % change per year (95% CI)	Year of rate-change*	Subsequent % change per year (95% CI)	Direction of change†	Estimate in 2014‡
Proportion of pneumonias with					
Blood or high-quality respiratory culture taken	−3.9 (−5.5 to −2.2)	2004	−1.1 (−1.9 to −0.3)	↓ ↓	0.62
Any potentially causative bacterial organism found	−3.4 (−5.6 to −1.1)	–		↓	0.05
Incidence of pneumonia with					
Blood or high-quality respiratory culture taken	2.1 (0.5 to 3.8)	2007	7.2 (6.3 to 8.2)	↑ ↑	1024
Any potentially causative bacterial organism found	3.8 (1.3 to 6.3)	–		↑	78
*S. pneumoniae*	−1.0 (−4.4 to 2.5)	–		↔	29
*S. aureus*	0.2 (−8.4 to 9.6)	–		↔	5
*E. coli*	9.8 (5.4 to 14.5)	–		↑	12
*Klebsiella* spp	12.3 (4.8 to 20.2)	–		↑	5
*P. aeruginosa*	8.6 (−5.6 to 25.0)	–		↔	3
*H. influenzae*	4.5 (−7.7 to 18.3)	–		↔	3
Other *Enterobacteriaceae*	10.6 (4.4 to 17.3)	–		↑	6
Anaerobes/*S. Milleri*	13.6 (2.8 to 25.6)	–		↑	3
Other Gram-positives	2.3 (−1.5 to 6.3)	–		↔	8
Other Gram-negatives	35.0 (−16.6 to 118.6)	–		↔	1
Mixed organisms	7.8 (0.8 to 15.4)	−		↑	4

All trends modelled using piecewise linear Poisson regression.

*Allowing change in trend over time if significant improvement over linear trend alone (p<0.05), with year of the change point chosen by profile likelihood^8^ (see Methods).

†Single arrows represent direction of change over the whole time period; paired arrows represent direction and relative size of change in the periods before and after the year when data suggested trend in rates changed (as in *).

‡Proportion/number of admissions, as estimated by the best-fitting model as presented in the table.

## Discussion

Hospital admissions for CAP are currently increasing by ∼9% per year among Oxfordshire adults and cannot be attributed simply to a growing, ageing population. This is not an artefact of repeat admissions for the same illness episode nor an increase in hospital-acquired pneumonia as trends persisted when excluding all readmissions and when excluding those with any previous recent hospitalisations, respectively. Additionally, trends in admissions for reasons other than pneumonia differ significantly to CAP (table 1 and figure 1C), suggesting that the increases are not merely a reflection of overall changes in admission numbers. The rise is also not explainable by ‘diagnosis-code-switching’ since hospitalisations for other, similar, diagnoses also increased during the same time period. However, the fact that pneumonia diagnoses increased at a faster rate after 2008 while COPD and other lower respiratory infections started to increase more slowly at around the same time suggests some limited diagnosis-switching could still be occurring.

An alternative explanation for the CAP increase is that more low-severity cases are presenting to hospital (where previously they were managed in primary care), and the decrease in length of stay and 30-day all-cause mortality over the latter part of the study period supports this hypothesis. However, the fact that readmissions for pneumonia increased over this time supports another plausible explanation for the shortening length of stay, namely an increased pressure for early discharge. The proportion of CAP admissions where blood or high-quality respiratory cultures were requested (a potential indicator of moderate-high severity), as well as CRP, also decreased in the latter part of the study; however urea and neutrophils stayed fairly stable, and the actual magnitude of the CRP change was very small, with most individuals still having clearly abnormal values. Therefore there does not seem to have been a clear shift towards admitting more low-severity cases, and despite the proportion of pneumonia cases warranting blood/respiratory cultures decreasing, the actual numbers of these cases (and therefore burden of CAP judged sufficiently severe to warrant requesting them) has continued to increase.

The main study strength lies in the use of linked electronic health records, which allow for triangulation of frequently used but arguably subjective data such as diagnostic codes, against more reliably objective data such as laboratory tests. Accuracy and completeness of diagnostic coding has changed over time^11^ as hospitals have adjusted to Payment by Results^16^ and to the publication of standardised mortality ratios, so relying on coding alone carries inherent risks. Although we have not attempted to measure the accuracy of the primary coding used in our hospitals, previous studies suggest it is sufficient for epidemiological analysis.^11^ ^17^ ^18^ We have, however, considered ‘diagnostic-code-switching’ in detail, as well as used only primary codes, which are subject to regular national audit.^19^ Furthermore, linkage to national mortality data provides a more accurate outcome measure than the inhospital mortality used in most studies based on administrative data.^20^

However, this strength is also the main limitation, since we are unable to investigate underlying mechanisms or specific factors, for example, specimen quality, in more detail. The ethically approved database does not contain general practice data, so we cannot assess pneumonia incidence in general practice, although other studies have done this.^21^ We also cannot identify whether gradual changes were made in referral practice during the study period, although there was no evidence of a major impact of the reorganisation of out-of-hours service in 2004. Even within our hospital data, two key tests routinely used to diagnose and assess pneumonia were not available, namely chest X-rays and blood gases, neither were other clinical data such as blood pressure and respiratory rate. While these would have given additional information, the lack of clinically relevant changes in other important severity biomarkers suggests they would be unlikely to produce substantially different results. Other potentially confounding factors that cannot be accounted for in any study using secondary data include those caused by changes in organisational culture or not recorded in the hospital systems; however, we could not identify any major changes in healthcare provision that co-occurred with the increase in pneumonia trend from 2008. Our study is in a single hospital group, and a recent study investigating CAP in people over 65 years of age in the UK community^21^ found an unexpectedly high overall rate in the South Central region (which our hospitals serve), for reasons which are unclear, so further studies are needed to explore the generalisability of these findings. However, this may be challenging with the routinely collected data available, as to our knowledge there is only one other UK hospital group with a comparable database linking admissions to laboratory tests.

In the USA, sepsis has been shown to be a likely candidate for diagnosis-switching, leading to the appearance of falling pneumonia rates between 2003 and 2009,^5^ which may not be genuine. However it is not the cause of the pneumonia increases we report here, since it is still relatively uncommon in our hospitals (<200 primary diagnoses in relevant specialties in 2014), and also displays an increasing trend. A more recent US study^22^ which counted sepsis diagnoses together with pneumonia found an overall increase between 1993 and 2011, while in Denmark, there was a large national increase in the pneumonia hospitalisation rate between 1997 and 2011.^23^ However, unlike our analysis, all these studies used diagnosis codes in isolation. We were not able to identify other large population-based studies, somewhat surprisingly given the importance of pneumonia as a cause of admission.

Our findings raise two major questions—first what is the cause for the observed increases in CAP, and second, regardless of cause, what should we do about it? We have investigated several potential causes, but further possibilities include changes in levels of deprivation, an increase in comorbidities not captured within our data (given the confounding of the Charlson comorbidity score by coding depth (see online supplementary figures S1 and S2)) and reduced access to out-of-hours primary care leading people to come directly to hospital. Nevertheless, it is likely that multiple factors are contributing to the increase, rather than there being a single cause. Historically, pneumonia has been considered predominantly bacterial in aetiology, with *S. pneumoniae* the leading organism.^24^ ^25^ However, we found a causative organism relatively rarely, in only 5% of cases in 2014 (8% where a culture was performed), potentially because of emphasis on early antibiotic initiation in emergency admissions,^26^ though also likely due to inherent difficulties in isolating organisms using culture-based methods. Vaccination programmes^27^ have significantly reduced the incidence of invasive pneumococcal disease in southern England^28^ ^29^ (although not everywhere^4^), but *S. pneumoniae* was still the most common causative organism found. However, its dominance is decreasing, and other organisms, particularly *Enterobacteriaceae* such as *E. coli* and *Klebsiella* spp are becoming more prevalent. This is particularly concerning given their high rates of resistance to empirical antibiotics commonly recommended for CAP.^30^ ^31^ Although these pathogens have traditionally been viewed as associated with hospital-acquired pneumonia, we found similar CAP trends in patients without any recent previous hospital exposure, and specimens were taken very close to admission, suggesting this is highly unlikely to be driven by misclassified hospital-acquired pneumonia. There were no changes in hospital procedures regarding specimen collection processing that could have caused this: in any case such changes would be associated with an immediate incidence change rather than the gradual year-on-year increase we observed.

Respiratory tract infections are the most common indication for hospital antibiotic prescribing in the UK, accounting for over 30% of prescriptions.^32^ Thus CAP increases will drive increases in antibiotic prescribing and, unaddressed, hinder our best attempts to reduce antibiotic use, a key priority given increasing antimicrobial resistance. Animal studies suggest that antibiotic-driven microbiota depletion can impair subsequent responses to influenza^33^ and bacterial challenges,^34^ raising the intriguing possibility that increasing use of antibiotics to treat presumed bacterial infections could actually be having the unintended consequence of increasing their incidence. Further respiratory microbiome studies could investigate this hypothesis in humans. We are unable to assess whether non-microbiologically confirmed CAP was actually viral with the data available, and while H1N1 pandemic influenza may have been partially responsible for increases in 2009/2010, this would not explain the ever-increasing trend. Developing rapid point-of-care diagnostics to identify viral versus bacterial aetiology will be crucial to support the clinician in balancing the timely administration of antibiotics (emphasised by national^26^ and international^35^ guidelines), against reducing unnecessary antibiotic prescriptions. A better understanding of short-course or tailored antibiotic durations may also assist in reducing antibiotic exposure.

In summary, this study suggests that rather than discounting increases in pneumonia admissions as simply indicative of a growing, ageing population, or an artefact of documentation or healthcare-seeking behaviour, we should be actively searching for the reasons behind the increase, as well as planning our healthcare systems appropriately—for the increase in admissions—and to mitigate the potential ‘collateral damage’ of antimicrobial resistance that may otherwise be seen as more and more patients are treated for pneumonia.

## Supplementary Material

Web supplement
