# Supplementary material for: Increasing burden of community-acquired pneumonia leading to hospitalisation, 1998–2014
Source: Thorax. 2016 Feb 17;71(6):535–42. doi: 10.1136/thoraxjnl-2015-207688 (PMC4893127; doi:10.1136/thoraxjnl-2015-207688)
Supplement: Web supplement [file thoraxjnl-2015-207688-s1.pdf]

SUPPLEMENTARY MATERIAL

(A) Further inclusion criteria and categorisation of ICD-10 diagnostic codes

Pneumonia**:**

A310	Pulmonary mycobacterial infection
A420	Pulmonary actinomycosis
A481	Legionnaires' disease
B012	Varicella pneumonia
B052	Measles complicated by pneumonia
B250	Cytomegaloviral pneumonitis
B59	Pneumocystosis
J100	Influenza with pneumonia, other influenza virus identified
J110	Influenza with pneumonia, virus not identified
J128	Other viral pneumonia
J129	Viral pneumonia, unspecified
J13	Pneumonia due to Streptococcus pneumoniae
J14	Pneumonia due to Haemophilus influenzae
J150	Pneumonia due to Klebsiella pneumoniae
J151	Pneumonia due to Pseudomonas
J152	Pneumonia due to staphylococcus
J153	Pneumonia due to streptococcus, group B
J154	Pneumonia due to other streptococci
J155	Pneumonia due to Escherichia coli
J156	Pneumonia due to other aerobic Gram-negative bacteria
J157	Pneumonia due to Mycoplasma pneumoniae
J158	Other bacterial pneumonia
J159	Bacterial pneumonia, unspecified
J168	Pneumonia due to other specified infectious organisms
J172	Pneumonia in mycoses
J180	Bronchopneumonia, unspecified
J181	Lobar pneumonia, unspecified
J182	Hypostatic pneumonia, unspecified
J188	Other pneumonia, organism unspecified
J189	Pneumonia, unspecified
J690	Pneumonitis due to food and vomit
J850	Gangrene and necrosis of lung
J851	Abscess of lung with pneumonia

Chronic obstructive pulmonary disease:

J40	Bronchitis, not specified as acute or chronic
J410	Simple chronic bronchitis
J42	Unspecified chronic bronchitis
J431	Panlobular emphysema
J432	Centrilobular emphysema
J438	Other emphysema
J439	Emphysema, unspecified
J441	Chronic obstructive pulmonary disease with acute exacerbation, unspecified
J448	Other specified chronic obstructive pulmonary disease
J449	Chronic obstructive pulmonary disease, unspecified

Other lower respiratory tract infections

J200	Acute bronchitis due to Mycoplasma pneumoniae
J208	Acute bronchitis due to other specified organisms
J209	Acute bronchitis, unspecified
J219	Acute bronchiolitis, unspecified
J22	Unspecified acute lower respiratory infection
J852	Abscess of lung without pneumonia

Nonspecific viral infections:

B349	Viral infection, unspecified

Sepsis:
A021	Salmonella sepsis
A392	Acute meningococcaemia
A394	Meningococcaemia, unspecified
A400	Sepsis due to streptococcus, group A
A401	Sepsis due to streptococcus, group B
A402	Sepsis due to streptococcus, group D
A403	Sepsis due to Streptococcus pneumoniae
A408	Other streptococcal sepsis
A409	Streptococcal sepsis, unspecified
A410	Sepsis due to Staphylococcus aureus
A411	Sepsis due to other specified staphylococcus
A412	Sepsis due to unspecified staphylococcus
A414	Sepsis due to anaerobes
A415	Sepsis due to other Gram-negative organisms
A418	Other specified sepsis
A419	Sepsis, unspecified
R572	Septic shock

Skin/soft tissue infections:
L030	Cellulitis of finger and toe
L031	Cellulitis of other parts of limb
L032	Cellulitis of face
L033	Cellulitis of trunk
L038	Cellulitis of other sites
L039	Cellulitis, unspecified
L088	Other specified local infections of skin and subcutaneous tissue
L089	Local infection of skin and subcutaneous tissue, unspecified
M7112	Other infective bursitis, Upper arm
M725	Necrotizing fasciitis
M7250	Necrotizing fasciitis, Multiple sites
M7251	Necrotizing fasciitis, Shoulder region
M7252	Necrotizing fasciitis, Upper arm
M7253	Necrotizing fasciitis, Forearm
M7255	Necrotizing fasciitis, Pelvic region and thigh
M7256	Necrotizing fasciitis, Lower leg
M7257	Necrotizing fasciitis, Ankle and foot
M7258	Necrotizing fasciitis, Other
M7259	Necrotizing fasciitis, Site unspecified
M726	Necrotizing fasciitis
M7263	Necrotizing fasciitis, Forearm
M7266	Necrotizing fasciitis, Lower leg

Upper respiratory infections:
A370	Whooping cough due to Bordetella pertussis
A379	Whooping cough, unspecified
J00	Acute nasopharyngitis [common cold]
J010	Acute maxillary sinusitis
J011	Acute frontal sinusitis
J013	Acute sphenoidal sinusitis
J018	Other acute sinusitis
J019	Acute sinusitis, unspecified
J020	Streptococcal pharyngitis
J028	Acute pharyngitis due to other specified organisms
J029	Acute pharyngitis, unspecified
J030	Streptococcal tonsillitis
J038	Acute tonsillitis due to other specified organisms
J039	Acute tonsillitis, unspecified

J040 Acute laryngitis
J041 Acute tracheitis
J042 Acute laryngotracheitis
J050 Acute obstructive laryngitis [croup]
J051 Acute epiglottitis
J068 Other acute upper respiratory infections of multiple sites
J069 Acute upper respiratory infection, unspecified
J320 Chronic maxillary sinusitis
J321 Chronic frontal sinusitis
J322 Chronic ethmoidal sinusitis
J323 Chronic sphenoidal sinusitis
J328 Other chronic sinusitis
J329 Chronic sinusitis, unspecified
J36 Peritonsillar abscess
J390 Retropharyngeal and parapharyngeal abscess
J391 Other abscess of pharynx

Lower urinary tract infections:
N300 Acute cystitis
N308 Other cystitis
N309 Cystitis, unspecified
N340 Urethral abscess
N342 Other urethritis
N390 Urinary tract infection, site not specified
O233 Infections of other parts of urinary tract in pregnancy
O234 Unspecified infection of urinary tract in pregnancy
O239 Other and unspecified genitourinary tract infection in pregnancy
O862 Urinary tract infection following delivery

Gastroenteritis:
A010 Typhoid fever
A011 Paratyphoid fever A
A014 Paratyphoid fever, unspecified
A020 Salmonella enteritis
A028 Other specified salmonella infections
A029 Salmonella infection, unspecified
A044 Other intestinal Escherichia coli infections
A045 Campylobacter enteritis
A046 Enteritis due to Yersinia enterocolitica
A048 Other specified bacterial intestinal infections
A049 Bacterial intestinal infection, unspecified
A059 Bacterial foodborne intoxication, unspecified
A081 Acute gastroenteropathy due to Norwalk agent
A083 Other viral enteritis
A084 Viral intestinal infection, unspecified
A085 Other specified intestinal infections
A09 Other gastroenteritis and colitis of infectious and unspecified origin
A099 Gastroenteritis and colitis of unspecified origin

All infections (excluding pneumonia):
Available on request

All non-infections:
Available on request

(B) – Microbiology samples included and organisms cultured from them defined as plausibly causative for pneumonia or excluded

Specimens included[26]: blood for culture, pleural fluid, and bronchoalveolar lavage

Organisms cultured from these specimens included as plausibly causative for pneumonia, or excluded completely (i.e. test result counted as negative).

Specifically included as plausibly causing pneumonia (n)	Specifically excluded (i.e. test counted as negative)
Streptococcus pneumoniae (480)<br>***Escherichia coli*** (117)<br>***Staphylococcus aureus*** (52)<br>***Methicillin resistant staphylococcus aureus*** (25)<br>***Haemophilus influenzae*** (40)<br>***Pseudomonas aeruginosa*** (35)<br><br>***Klebsiella* species*:***<br>*Klebsiella pneumoniae* (40)<br>*Klebsiella oxytoca* (6)<br>*Klebsiella* species (1)<br><br>**Other *Enterobacteriaceae:***<br>*Citrobacter braakii* (1)<br>*Citrobacter freundii* (1)<br>*Citrobacter* species (2)<br>Coliforms (23)<br>*Enterobacter aerogenes* (2)<br>*Enterobacter cloacae* (9)<br>*Morganella morganii* (1)<br>Nonfermenting coliform (1)<br>*Proteus mirabilis* (19)<br>*Proteus* species (2)<br>*Proteus vulgaris* (5)<br>*Providencia* species (1)<br>*Salmonella* species (1)<br>*Serratia marcescens* (2)<br>*Yersinia enterocolitica* (1)<br><br>**Anaerobes*/Streptococcus milleri*:**<br>*Bacteroides fragilis* (2)<br>*Bacteroides intermedius* (1)<br>*Bacteroides uniformis* (1)<br>*Clostridium* species (5)<br>Mixed anaerobes isolated sensitive to metronidazole. (5)<br>*Streptococcus anginosus* (*milleri* group) (2)<br>*Streptococcus constellatus* (*milleri* group) (2)<br>*Streptococcus intermedius* (*milleri* group) (1)<br>*Streptococcus milleri* (7)<br><br>**Other Gram-positives:**<br>Alpha haemolytic *streptococci* (64)<br>Beta haemolytic *streptococci* (1)<br>Group A *streptococci* (24)<br>*Streptococci* (8)<br>*Streptococci,* beta haemolytic group B (5)<br>*Streptococci,* beta haemolytic group C (5)<br>*Streptococci,* beta haemolytic group G (12)<br>*Streptococcus bovis* (1)<br>*Streptococcus dysgalactiae* (serogroup C/G) (1)<br>*Streptococcus equisimilis* (1)<br>*Streptococcus gallolyticus* (*strep.bovis* biotype I) (1)<br>*Streptococcus mitis* (8)<br><br>**Other Gram-negatives:**<br>*Capnocytophaga canimorsus* (1)<br>*Eikenella corrodens* (1)<br>*Haemophilus* species (3)<br>*Neisseria meningitidis* (1)	*Acinetobacter baumannii*<br>*Acinetobacter lwoffii*<br>*Acinetobacter radioressistens*<br>*Acinetobacter* species<br>*Actinomyces bovis*<br>Aerobic spore bearer<br>*Aerococcus* species<br>*Aerococcus viridans*<br>*Bacillus* species<br>*Candida*<br>*Candida albicans*<br>*Candida glabrata*<br>*Candida* species<br>*Coagulase negative staphylococcus*<br>Colonies<br>*Corynebacterium afermentans*<br>*Corynebacterium amycolatum*<br>*Corynebacterium jeikeium*<br>*Corynebacterium* species<br>*Corynebacterium striatum*<br>Diphtheroids<br>*Enterococci*<br>*Enterococcus faecalis* group D<br>*Enterococcus faecium* group D<br>*Enterococcus* species<br>*Gemella haemolysans*<br>*Gemella morbillorum*<br>*Lactobacillus casei*<br>*Lactobacillus* species<br>*Listeria monocytogenes*<br>*Micrococci*<br>*Micrococcus luteus*<br>*Micrococcus* species<br>*Moraxella catarrhalis (branhamella catarrhalis)*<br>*Moraxella species*<br>*Neisseria species*<br>Non lactose fermenting Gram negative rods<br>*Pasteurella multocida*<br>*Propionibacterium acnes*<br>*Propionibacterium* species<br>*Pseudomonas luteola*<br>*Pseudomonas* species<br>*Pseudomonas stutzeri*<br>*Rhodococcus* species<br>*Rothia dentocariosa*<br>*Staphylococcus capitis*<br>*Staphylococcus epidermidis*(CONS)<br>*Staphylococcus haemolyticus*<br>*Stenotrophomonas maltophilia*

Supplementary Methods

For piecewise linear Poisson regression, data was aggregated by UK financial year (Apr-Mar) and separate models were built for each potential change-point between 2000 and 2012 inclusive, using two linear piecewise linear functions (with the standard Poisson log link). The model which produced the lowest deviance goodness-of-fit statistic was chosen as the best two-piece model (a standard profile likelihood[8] approach). This model was then compared to the model with a single linear trend using a chi-squared test of difference in deviance, and the two-piece model chosen if it was a significantly ($p<0.05$) better fit.

Piecewise linear median regression was conducted similarly, with change-point model selection using the pseudo-R-squared statistic, and the best two-piece model chosen over the single trend model if an F-test of before-and-after trends showed a significant ($p<0.05$) difference.

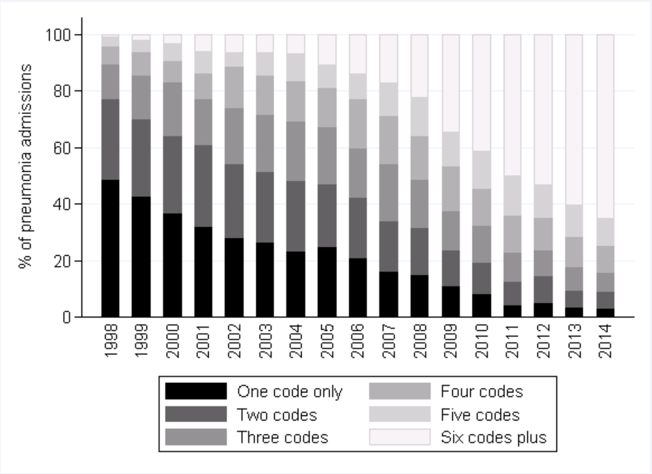

Supplementary Figure 1. Coding depth – number of diagnostic codes recorded per CAP admission
Note: electronic recording of secondary diagnosis codes was introduced in January 2006.

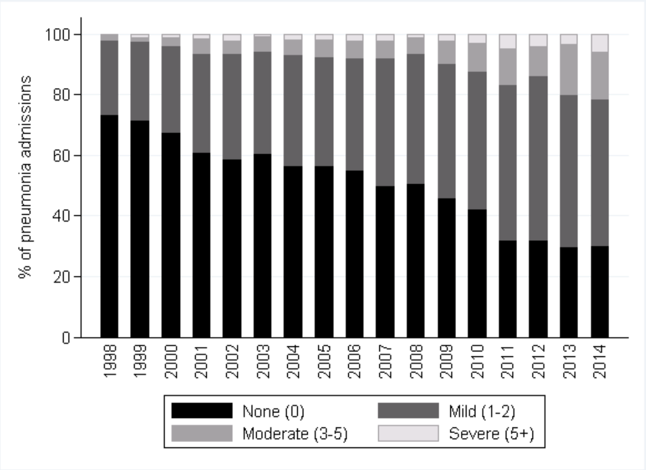

Supplementary Figure 2. Charlson severity score per CAP admission

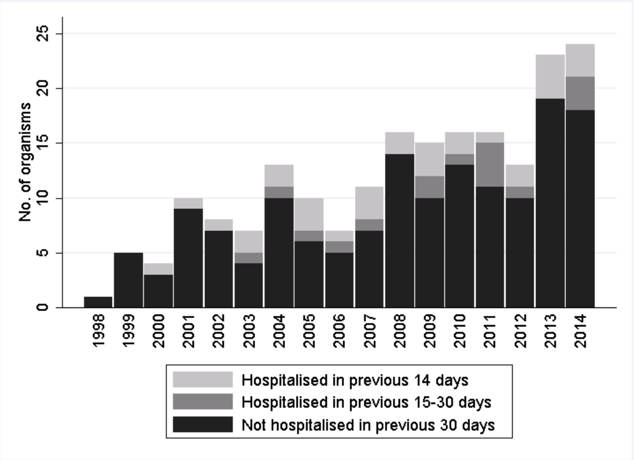

Supplementary Figure 3. Prior recent hospitalisation of patients with *Enterobacteriaceae* cultured
